# Quantitative Assessment of the Influence of Tensile Softening of Concrete in Beams under Bending by Numerical Simulations with XFEM and Cohesive Cracks

**DOI:** 10.3390/ma15020626

**Published:** 2022-01-14

**Authors:** Ireneusz Marzec, Jerzy Bobiński

**Affiliations:** Department of Engineering Structures, Faculty of Civil and Environmental Engineering, Gdansk University of Technology, Narutowicza 11/12, 80-233 Gdansk, Poland

**Keywords:** concrete, size effect, fracture energy, cohesive crack, XFEM, three-point bending

## Abstract

Results of the numerical simulations of the size effect phenomenon for concrete in comparison with experimental data are presented. In-plane geometrically similar notched and unnotched beams under three-point bending are analyzed. EXtended Finite Element Method (XFEM) with a cohesive softening law is used. Comprehensive parametric study with the respect to the tensile strength and the initial fracture energy is performed. Sensitivity of the results with respect to the material parameters and the specimen geometry is investigated. Three different softening laws are examined. First, a bilinear softening definition is utilized. Then, an exponential curve is taken. Finally, a rational Bezier curve is tested. An ambiguity in choosing material parameters and softening curve definitions is discussed. Numerical results are compared with experimental outcomes recently reported in the literature. Two error measures are defined and used to quantitatively assess calculated maximum forces (nominal strengths) in comparison with experimental values as a primary criterion. In addition, the force—displacement curves are also analyzed. It is shown that all softening curves produce results consistent with the experimental data. Moreover, with different softening laws assumed, different initial fracture energies should be taken to obtain proper results.

## 1. Introduction

During a cracking process in concrete so called fracture process zone is created. Its size is not negligible comparing to specimen’s dimensions. As a consequence, the behavior of concrete structures exhibits strong size effects, i.e., small elements have a greater nominal strength than large ones. The size effect depends also on the geometry on the boundaries, e.g., unnotched versus notched beams. All mentioned difficulties cause problems in proper determination of concrete material properties or performing advanced numerical simulations. There is a variety of alternative approaches and there is still no consensus in describing the fracture in concrete. The proper description of cracks is, therefore, crucial in obtaining physically admissible results in simulations of concrete [1,2] and reinforced concrete specimens [3,4,5,6]. Within continuum mechanics, cracks can be defined in a smeared sense [7,8] or as a discrete one with cohesive elements [9,10] or based on Extended Finite Element Method (XFEM) [11,12,13,14,15,16]. In advanced formulations these two approaches can be coupled [17,18].

A very important issue in the numerical description of cracks in concrete is a realistic definition of a constitutive law for concrete in tension. The behaviour of concrete under uniaxial tension is strongly nonlinear. Initially concrete behaves as a linear elastic material (approximately up to 90% of the tensile strength) followed by a hardening phase. After reaching the tensile strength a gradual decrease of the tensile strength starts (so-called quasi-brittle behaviour). When a mathematical description of concrete under tension is formulated, a hardening region before the peak is usually neglected and the material behaviour is assumed to be linearly elastic up to the peak. In order to the describe the softening phase (after the peak) several alternative formulations can be used. The most popular and most commonly used are the linear, bilinear, and exponential (or quasi exponential) softening curves.

The simplest possibility to define the softening of concrete in tension is to assume a linear softening relationship. Unfortunately, this approach generally gives wrong (non-physical) results and therefore it should be avoided in advanced numerical simulations of concrete specimens. Realistic results can be obtained by adopting the bilinear softening law [19] with two sections with different inclination (softening modulus). The additional issue here is the definition of the kink point. Petterson [19] assumed the yield stress at the kink point $\sigma_{k}$ equal to one third of the tensile strength $f_{t}$. The displacement at the kink $\kappa_{k}$ point was taken as 0.22 of the ultimate displacement $\kappa_{u}$ ultimate displacement (when yield stress is equal to zero). Wittman et al. [20] proposed the kink point at the level of 25% of the tensile strength $f_{t}$. The CEB-90 model code [21] for normal strength concrete took the ratio between the $\sigma_{k}$ and $f_{t}$ as 0.15. Bažant [22] stated the kink point should be defined between the 0.15 and 0.33 of the tensile strength $f_{t}$. Park et al. [23] numerically analysed several different definitions of the kink point location with the $\sigma_{k}/f_{t}$ ratio between the 0.18 and 0.42. They implemented a cohesive zone model into the Abaqus software. The second family of softening curves utilises exponential function starting from almost classical formulation (Gopalaratnam and Shah [24]) to very complex relationships (Reinhardt et al. [25], Chen and Su [26]).

Tang and Chen [27] compared numerically bilinear softening curve by Wittman et al. [20], exponential relationship by Reinhardt et al. [25] and exponential formula by Chen and Su [26] with experiments. They found the best agreement for normal strength concrete was achieved with the curve by Reinhardt et al. [25], while the formula proposed by Chen and Su [26] gave the best results for the high strength concrete. It should be noted, however, that the differences were minimal. Kumar and Barai [28] numerically simulated concrete compact tension specimen with different softening curves. The best agreement was obtained with the exponential curve by Reinhardt et al. [25] and bilinear curve by Wittmann et al. [20] while the linear softening relationship gave the worst results. Dong et al. [29] tested experimentally two series of notched concrete beams under three-point bending with different notch heights and constant height (B-series) and with different heights and constant notch size (L-series). Next, they run numerical simulations using bilinear softening law proposed by Petterson [19]. Alternatively, the size effects for quasi-brittle materials can be described using the fractal approach to the mechanics of material e.g., within cohesive crack model [30]. The in-depth review of investigation and application of fractal theory in cement-based materials was presented by Wang et al. [31].

Recently some extensive research programs were executed to improve the understanding of concrete fracture. Hoover et al. [32] examined unnotched and notched concrete beams under three-point bending. Four different geometrically similar beam sizes of five different notch to depth ratios were analyzed. More than one-hundred specimens were tested. Based on experimental data initial and total fracture energies were determined [33]. The identical beams (with the respect to geometry data) were tested by Çağlar and Şener [34]. In total, 80 specimens were casted. The main difference comparing to Hoover et al. [32] research was the direction of casting: instead of the horizontal position, beams were casted vertically. In addition, the support rotations were measured. Independently, similar experiments were conducted by Grégoire et al. [35]. They examined geometrically similar unnotched and notched beams of four different sizes and three different notches to depth ratios. In total, 34 specimens were tested.

Experimentally obtained peak loads were later used to verify different theoretical size effect laws. Hoover and Bažant [36] compared experimental results from [32] with improved universal size effect law (USEL). A very good agreement was achieved. Hu et al. [37] presented an extensive comparison of different formulations of size effect laws and boundary effect model against experimental results of Hoover et al. [32]. Çağlar and Şener [34] on the basis of their experimental results verified universal size effect law (USEL) proposed by Hoover and Bažant [36] and boundary effect model of Duan et al. [38,39]. They stated that Bažant’s Type I size-effect law is reasonably good for beams with small notches, and Type II size-effect law fits favourably for beams with deep notches. They also observed that boundary effect model provides good comparison for unnotched beams. Grégoire et al. [35] compared the experimental outcomes with USEL proposed by Bažant and Yu [40] and found consistent match between them.

Several researchers performed numerical simulations and compared their results with experimental outcomes cited above. Hoover and Bažant [41] used the crack band model defined as an isotropic damage model with an equivalent strain based on Mazar’s proposal [42] and a bilinear softening law. They also tried to fit experimental data using a linear or an exponential softening law, but without a success. They presented, however, no results with exponential or exponential softening curves to support this statement. Lorentz [43] formulated a nonlocal gradient model consistent with cohesive fracture. To describe the post-peak behavior of the material he proposed a combination of a linear polynomial and an exponential function with two parameters. Grégoire et al. [35] took the isotropic damage constitutive law with Mazar’s [42] equivalent strain, an exponential softening and integral non-local regularization method. They obtained a good agreement with experiments of the middle-size specimens, but much worse results were achieved of the smallest or largest beams. Feng and Wu [44] used phase-field regularized cohesive zone model with a very small length scale to simulate notched and unnotched concrete beams experimentally tested by Gregoire et al. [35] and Hoover et al. [32]. In the softening regime, they adopted the exponential curve proposed by Reinhardt et al. [25]. Barbat et al. [45] used a local version of an isotropic damage constitutive law with an exponential law to simulate both experimental campaigns by Gregoire et al. [35] and Hoover et al. [32]. They reported a good agreement between numerical simulations and experimental outcomes. Parrilla Gomez et al. [46] simulated again these two experimental series with a model of graded damage with Thick Level Set (TLS) method. Zhang et al. [47] used localizing gradient damage model to numerically reproduce both Hoover et al. [32] and Grégoire et al. [35] experiments. Wosatko et al. [48] examined the behaviour of two constitutive laws: the consistency viscoplasticity and the gradient damage model against the experimental outcomes of Grégoire et al. [35] experiment. Marzec and Bobiński [49] adopted elasto-plastic model with Rankine criterion in tension enriched by an integral non-local regularisation approach to simulate Hoover et al. [32] test.

Havlásek et al. [50] used an isotropic damage model with an equivalent strain corresponding to the Rankine criterion with round-off in multiaxial tension region. An exponential curve was adopted in the softening regime. The integral non-local theory was used as a regularisation method. They studied standard and distance based averaging methods. In the second approach the characteristic length in points lying near the boundary decreased with decreasing the distance to the specimen’s edge. They found that the distance-based method was able to reproduce the experimental results well for all notch lengths and beam sizes, while standard averaging significantly overestimated the nominal strength of the small notched beams. The distance-based approach by decreasing the characteristic length results in reducing the localisation zone width. As a consequence, the fracture energy in the material points lying in the boundary layer is also reduced. Its idea can be related to nonlocal boundary layer model proposed by Bažant et al. [51] to overcome numerical problems in treating boundaries in fracture analysis when integral non-local averaging algorithm is used as a regularisation technique. Vořechovský [52] used the similar idea of the ‘weakened boundary layer’ to explain experimental results of van Vliet and van Mier [53,54]. They tested the dog-bone specimens of different sizes subjected to uniaxial tension. Surprisingly the smallest specimen turned out not to be the strongest one (with the respect to the nominal strength).

The above review shows there is no clear consensus when modeling the softening phase of cracks in concrete in mode I. Therefore the aim of the paper is to examine the performance of the discrete cohesive crack model equipped with different softening curve definitions in simulating the size effect phenomenon in concrete beams under bending to verify the opinion of the superiority of the bilinear softening curves over alternative formulations. Contrary to smeared crack formulations used in simulation studies mentioned above, a discrete approach is used here within Extended Finite Element Method (XFEM). The attention is paid to the ability of different softening laws: bilinear, exponential, and based on rational Bezier curve to properly reflect the experimental outcomes. The influence of the reduction of the fracture energy in the boundary layer is also examined. Error measures are introduced to quantitatively estimate calculated peak loads. In none of the papers cited above such quantitatively assessment has not been done. Obtained force—crack mouth open displacement (CMOD) curves are compared with experimental diagrams only qualitatively (visually). Analyses determining the best initial fracture energies are performed. The main goal of presented numerical results is the comparison with experiments, therefore no fitting to different size effect laws is done here.

The paper is organized as follows. Section 2 outlines the main ideas of the paper. Section 3 presents the experimental research performed by Hoover et al. [32]. Their results serve as a verification data to rate material parameters used in FE-calculations. Section 4 provides information about the formulation of the eXtended Finite Element Method and the constitutive law used to describe a discrete crack. Some details of the implementation issues are also added. Numerical results are extensively presented in Section 5. The final conclusions and future plans are listed in Section 6.

## 2. Significance of Research

Current research, concerning a series of geometrically scaled concrete beams with and without notch under three-point bending, is an extension of our previous investigations of concrete fracture properties [49]. The attention is put on influence of different softening curves definition and material parameters in concrete (i.e., fracture energy and initial fracture energy) on specimen strength under bending. This knowledge is important to better understand a fracture phenomenon and to provide reliable numerical tool to describe the size effect in concrete members. Thus, the main objective of this study that also represent its novelty is detailed and quantitative (not only qualitative) assessment of efficiency of different softening curves in numerical prediction of size effect for concrete beams under bending.

## 3. Experiment by Hoover

### 3.1. Geometry of the Beams

**Figure 1:**
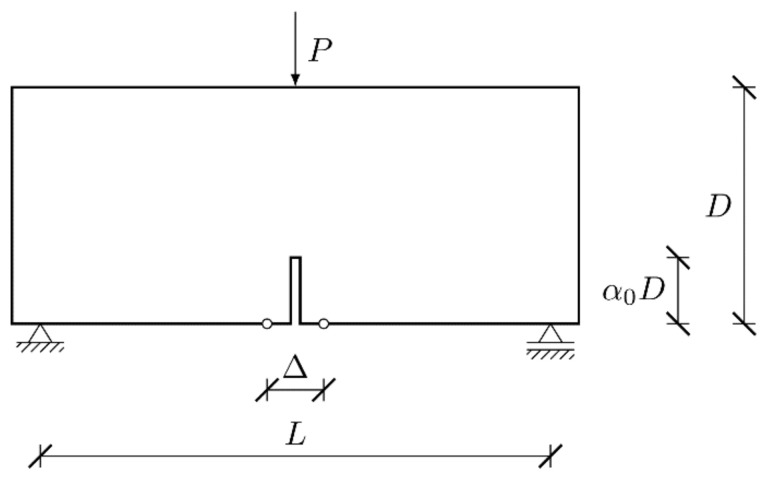
Geometry of the beam and imposed boundary conditions.

Fracture process in one of the fundamental phenomenon in concrete. The adequate description is a crucial issue and it is essential to formulate a proper physically meaningful constitutive law. There have been several experimental campaigns on fracture in concrete. One of the recently reported research was conducted by Hoover et al. [32]. They examined 128 unnotched and notched concrete beams under three-point bending. The geometry of a beam is shown at Figure 1. Four different sizes with five notch dimensions were analysed. The beam height D was taken as 500, 215, 93 and 40 mm for a huge, large, medium, and small specimen, respectively. The total length of the beam was 1200, 516, 223.2 and 96 mm for a huge, large, medium, and small specimen, respectively. The notch to depth ratio $\alpha_{0}$ (relative length of a notch with the respect to the beam’s height D) was 0.0 (no notch), 0.025, 0.075, 0.15 and 0.30. In total, 18 geometries were defined (beams with the height equal to D = 93 mm and D = 40 mm with $\alpha_{0}\;$= 0.025 were not cast). The span length L was equal to 2.167D for all beams. The thickness was set to B = 40 mm. The width of the notch was 1.5 mm. In addition, 36 companion samples were prepared to determine the compressive strength, modulus of rupture, Young’s modulus, and Poisson’s ratio. All specimens were cast within three hours from the same batch of concrete. They were kept in identical curing and environmental conditions. As a consequence, low scatter of results was achieved, and all beams had virtually the same material properties.

### 3.2. Experimental Results

All tests were carried out under opening displacement control. Two points at the bottom edge lying symmetrically with the respect to the vertical axis of symmetry of the beam were chosen and a gauge was placed. The gauge length was scaled with the beam size. Steel loading blocks with dimensions 60 × 40 × 40, 25.8 × 17.2 × 40, 11.1 × 7.4 × 40 and 4.8 × 3.2 × 40 mm for a huge, large, medium, and small specimen, respectively, were placed under the load and supports. The nominal strength $\sigma_{N}$ was calculated for all results:

(1) $$\sigma_{N}=\frac{3}{2}\cdot \frac{2.176P_{u}}{BD}$$

where $P_{u}$ is the maximum force. Table 1 presents mean nominal strengths, $\sigma_{N}^{Exp}$ for each beam geometry (after [30]). In this paper, the correction factor $C_{f}$ was calculated for each geometry. This factor considers real dimensions measured in experiments, usually different than nominal beam dimensions B and D. It is defined as:

(2) $$C_{f}=\frac{2}{3}\cdot \frac{\sigma_{N}^{Exp}BD}{2.176P_{u}^{Exp}}$$

where $P_{u}^{Exp}$ is the averaged maximum force for a given geometry (calculated from maximum force values given in [32]). Averaged maximum forces $P_{u}^{Exp}$ and correction factors $C_{f}$ are presented in Table 1. The correction factor of the unnotched medium beam (D = 93 mm) was rather unrealistic and it deviated significantly from other values (bold number in Table 1), therefore a value 1.0 was assumed instead.

## 4. Constitutive Laws

### 4.1. General Formulation

Cohesive cracks will be described here via Extended Finite Element Method (XFEM). This approach is based on a Partition of Unity concept [55]. It allows for adding extra terms to the standard Finite Element (FE) displacement field approximation for a better capture of a displacement discontinuities. The key point is to enrich only selected nodes with additional degrees of freedom (locally “near” a crack) and to remain the remaining part of the specimen standard. As the crack propagates during FE calculations, the number of enriched nodes increases dynamically. Using XFEM, cracks can pass through finite elements without any remeshing; they do not have to follow the edges of the elements.

**Figure 2:**
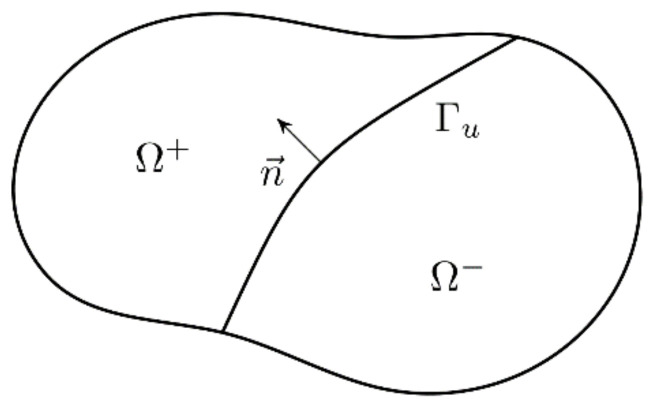
Body cut by a discontinuity.

The formulation used in this paper follows generally the classical idea presented by Wells and Sluys [11]. The only fundamental difference is the application of the shifted-basis enrichment (Zi and Belytschko [56]). In a body Ω cut by a discontinuity $\Gamma_{d}$ (Figure 2), the displacement field u in a point x can be calculated as (using a finite element format):

(3) $$u\left(x\right)=\underset{I\in N_{tot}}{\sum }N_{I}\left(x\right)a_{I}+\underset{I\in N_{enr}}{\sum }N_{I}\left(x\right)\left(\psi \left(x\right)-\psi \left(x_{I}\right)\right)b_{I}$$

where $N_{I}$ is a shape function in a node I, $N_{tot}$ is the set of all nodes, $N_{enr}$ is the set of enriched nodes (nodes of the elements cut by the crack), $a_{I}$ are standard displacements (in a node *I*), $b_{I}$ are enriched displacements in a node I and ψ denotes a (generalized) step function (a sign function) defined as:

(4) $$\psi \left(x\right)=\left\{\begin{array}{cc} 1 & x\in \Omega^{+}\\ -1 & x\in \Omega^{-} \end{array}\right.$$

In the original formulation [11], the Heaviside step function was used. This shift does not change the approximating basis, but it simplifies the formulation of the method. This enrichment is equal to zero in all elements not cut by a crack; as a consequence, only two types of finite elements have to be defined. Moreover, total displacements in nodes are equal to standard ones. The weak form of equilibrium, discretized equations, and the details of the finite element derivations can be found in Zi and Belytschko [56] and Tejchman and Bobiński [57].

### 4.2. Bulk Material Description

Within this approach two material laws have to be defined. The first one describes the behavior of the material in a solid (bulk) body. In uncracked continuum, a linear elastic constitutive relationship between strains ε and stresses σ is assumed:

(5) $$\sigma =D^{e}\varepsilon$$

where $D^{e}$ is a linear elastic material matrix. Assuming plane stress conditions, the matrix $D^{e}$ is calculated as:

(6) $$D^{e}=\frac{E}{1-\upsilon^{2}}\left[\begin{array}{ccc} 1 & \upsilon & 0\\ \upsilon & 1 & 0\\ 0 & 0 & 1-\upsilon \end{array}\right]$$

where E is Young’s modulus and ν denotes Poisson’s ratio.

### 4.3. Discrete Crack Definiton

The second constitutive relationship defines the behavior of the cohesive crack. A new crack segment can be created, if the Rankine criterion (plane stress case) is fulfilled:

(7) $$\max\left\{\sigma_{1},\;\sigma_{2}\right\}>f_{t}$$

where $\sigma_{1}$ and $\sigma_{2}$ are the principal stresses and $f_{t}$ is the tensile strength. This inequality is checked in all integration points in the finite element at the front of the crack tip. The crack grows if Equation (7) is true in at least one integration point. Due to a symmetry of the problem (three-point bending test) and isotropic and homogeneous material definition, a fixed vertical direction of the crack propagation is assumed. A new segment is defined from one element’s side to another one (crack tip cannot be placed inside a finite element). Segment end points cannot be placed at element’s vertices. For integration purposed a cracked element is divided into three sub-triangles with one-point Gauss quadrature, while two integration points are defined along the crack segment (Asferg et al. [58]).

Along a crack a cohesive traction vector t is defined (it is not a stress-free crack formulation). The traction vector t is related with displacement jumps 〚u〛. Both these quantities are defined in a local coordinate system and they have normal (index n) and tangential (index s) components. Due to the tensile dominated nature of the problem, the following loading function *f* is assumed:

(8) $$f\left({〚u〛}_{n},\kappa \right)={〚u〛}_{n}-\kappa$$

where κ is an internal variable, equal to the largest value of the normal displacement ${〚u〛}_{n}$ obtained during the loading history. Active loading occurs for f≥0 and unloading (reloading) is indicated by f<0. During active loading the normal traction force is equal to the yield traction $t_{y}$:

(9) $$t_{n}=t_{y}\left(\kappa \right)$$

**Figure 3:**
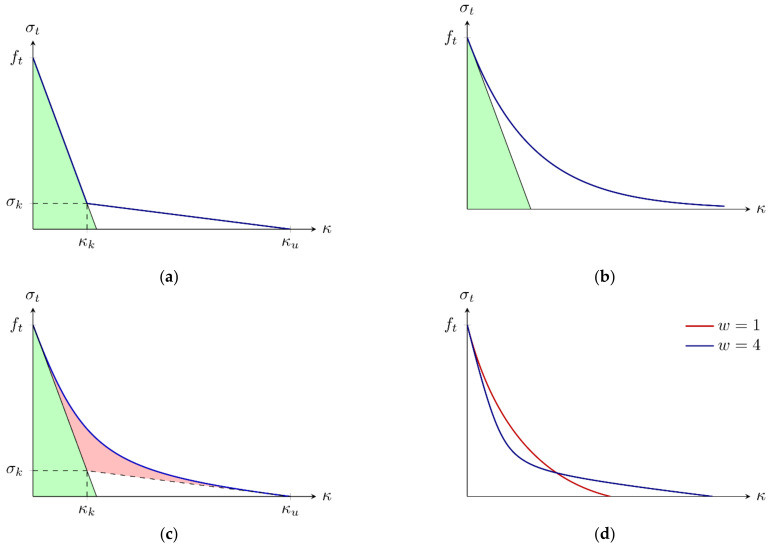
Softening curves: (**a**) bilinear; (**b**) exponential; (**c**,**d**) rational Bezier.

Three (basic) softening curves are used to calculate the yield traction $t_{y}$. First, a bilinear curve is defined (Figure 3a):

(10) $${\bar{t}}_{n}\left(\kappa \right)=\left\{\begin{array}{ll} f_{t}+\left({\bar{t}}_{k}-f_{t}\right)\frac{\kappa }{\kappa_{k}} & \kappa <\kappa_{k}\\ {\bar{t}}_{k}\frac{\kappa_{u}-\kappa }{\kappa_{u}-\kappa_{f}} & \kappa_{k}\leq \kappa <\kappa_{u}\\ 0 & \kappa \geq \kappa_{u} \end{array}\right.$$

where ${\bar{t}}_{k}$ and $\kappa_{k}$ are the traction and the value of the internal variable κ at the kink point, respectively, and $\kappa_{u}$ is the value of the of the internal variable (crack opening)  κ when the traction ${\bar{t}}_{n}$ is reduced to zero. Parameters $\kappa_{k}$ and $\kappa_{u}$ can be calculated as:

(11) $$\kappa_{k}=\frac{2G_{f}}{f_{t}^{2}}\left(f_{t}-{\bar{t}}_{k}\right)\;\mathrm{and}\;\kappa_{u}=\frac{2}{{\bar{t}}_{k}}\left(G_{F}+G_{f}\left(\frac{{\bar{t}}_{k}}{f_{t}}-1\right)\right)$$

Using the total fracture energy $G_{F}$ and the initial fracture energy $G_{f}$ (area under the initial tangent line from the peak point at Figure 3, marked as a green area). The equivalent relationship (in a continuum format) was used by Hoover and Bažant [41].

As a second alternative an exponential function is chosen (Figure 3b):

(12) $${\bar{t}}_{n}\left(\kappa \right)=f_{t}\exp\left(-\frac{f_{t}}{G_{F}}\kappa \right)$$

The area under this curve is equal to the total fracture energy $G_{F}$ and the ratio between the total fracture energy $G_{F}$ and the initial fracture energy $G_{f}$ is equal to 2 (it is a fixed value, it does not depend on the curve parameters $f_{t}$ and $G_{F}$).

The last proposal is formulated using Bezier rational curve based on the bilinear softening definition (Figure 3c,d) to allow the smooth transition between two segments (without a sudden change of the direction in the kink point). It is defined via two parametric equations:

(13) $$\kappa \left(t\right)=\frac{2\left(1-t\right)tw\kappa_{k}+t^{2}\kappa_{u}}{{\left(1-t\right)}^{2}+2\left(1-t\right)tw+t^{2}}\;\mathrm{and}\;{\bar{t}}_{n}\left(t\right)=\frac{{\left(1-t\right)}^{2}f_{t}+2\left(1-t\right)tw{\bar{t}}_{k}}{{\left(1-t\right)}^{2}+2\left(1-t\right)tw+t^{2}}$$

With a parameter t and a weight w attached to the kink point. The weight w controls the shape of the curve; for w = 0 a linear softening is obtained and for w  =  ∞ it coincides with the bilinear diagram (Figure 3a). Here the “total” fracture energy used to define the kink point on the underlying bilinear softening curve is denoted as $G_{B}$ and it is not equal to the total fracture energy $G_{F}$ (except for the case with w  =  ∞). The term $G_{F}-G_{B}$ can be interpreted as the area between the Bezier rational curve and the bilinear curve (marked as a red area in Figure 3c). The larger the value of the weight w is taken, the smaller difference $G_{F}-G_{B}$ is obtained.

Given a basic softening curve ${\bar{t}}_{n}$, the yield traction is defined as:

(14) $$t_{y}=D_{f}{\bar{t}}_{n}$$

where $D_{f}$ is a correction term calculated as:

(15) $$D_{f}=1-\exp\left(-\frac{d_{f}f_{t}}{G_{F}}\right)$$

With a drop factor $d_{f}$ (Cox [59]). The presence of the correction term $D_{f}$ improves the convergence in cases where transition between tension and compression occurs, resulting in sudden stiffness changes. With increasing the value of the drop factor $d_{f}$, the correction term $D_{f}$ goes to one and the original softening definition is recovered.

During unloading, the secant stiffness is used with a return to the original configuration (damage format):

(16) $$t_{n}=\frac{t_{y}}{\kappa }{〚u〛}_{n}$$

In compression the penalty stiffness $T_{n}$ is taken:

(17) $$T_{n}=\frac{d_{f}f_{t}^{2}}{G_{F}}$$

It is calculated as a derivative of the yield traction (Equation (14)) at κ=0.

In a tangential direction a linear dependence on the current yield traction is assumed:

(18) $$t_{s}=T_{s}\frac{t_{y}}{f_{t}}{〚u〛}_{s}$$

With the initial shear stiffness $T_{s}$. It ensures that the shear traction decreases to zero while the crack opens. This idea is close to the exponential softening postulated by Wells and Sluys [11].

### 4.4. Boundary Layer

**Figure 4:**
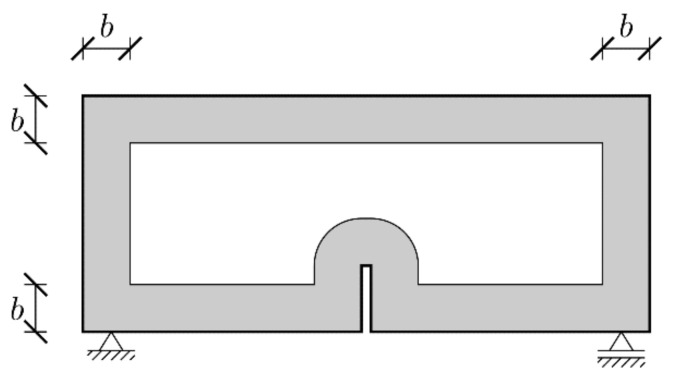
Geometry of the boundary layer zone.

In order to verify the necessity of introducing the weaker boundary layer postulated by some researchers to obtain physically consistent results (Vořechovský [52]) some extra simulations are carried out. Figure 4 presents the geometry of the weaker boundary layer zone of the width *b* along the specimen’s edges (including notch). Within this zone initial and total fracture energies are calculated as $\beta G_{f}$ and $\beta G_{F}$, where a reduction coefficient β is defined as:

(19) $$\beta \left(x\right)=\left\{\begin{array}{ll} \beta_{0}+\left(1-\beta_{0}\right)\frac{d\left(x\right)}{b} & d\left(x\right)<b\\ 1 & d\left(x\right)\geq b \end{array}\right.$$

Here $d\left(x\right)$ is the distance of the point x to the nearest edge and $\beta_{0}$ is the value of the reduction coefficient at the boundary. A linear decrease is assumed here, but other relationships, e.g., exponential one [50], can be also used. Although such reduction was not performed by Hoover and Bažant [41] and Lorentz [43], it was essential to obtained experimentally consistent results by Havlásek et al. [50].

### 4.5. Implementation

Numerical calculations have been performed using a commercial program Abaqus Standard [60]. Although it includes XFEM procedures, this implementation has some limitations. Only quadrilateral finite elements are allowed and there is no possibility to define user’s crack direction propagation criteria. Therefore, the Abaqus user-defined element procedure (UEL) is utilized to implement a finite element within XFEM. The independent module in Fortran 95 has been written to handle model data and needed subroutines. Within this module nodal coordinates, elements connectivity data, information from integration points are kept. This module is then called in for the UEL subroutine. Such approach gives the access to gathered model data from each finite element (it is not possible by default). The convergence criteria taken from Abaqus [60] are applied:

(20) $$r_{\max}\leq 0.01\tilde{q}\;\mathrm{and}\;c_{\max}\leq 0.01\Delta u_{\max}$$

where $r_{\max}$ is the largest residual in a balance force vector (right hand side vector), $\tilde{q}$ is an overall time-averaged value of all element force vectors and external loads, $c_{\max}$ stands for the largest correction (change between last two iterations) of the unknown displacements and $\Delta u_{\max}$ depicts the largest change of the unknown displacement in the increment.

The new crack segments can be created only in a converged configuration. Then, a restart procedure is applied, and a current increment is repeated to find a converged configuration again. A crack can be extended by one segment (in one finite element) only between two converged configurations. This procedure is repeated as long new crack segments are created in a current increment. If no new crack segments have been added, the next increment starts (after convergence). In order to implement this idea into Abaqus, an independent convergence algorithm has been developed. Information about residuum forces and displacement corrections is gathered (independently from Abaqus) in the Fortran module (it is transferred from user elements). One-node user elements with zero stiffness matrix and zero force vector are manually defined in an input file in nodes with imposed boundary conditions. These elements transfer information on defined displacement/boundary conditions to exclude appropriate degrees of freedom from convergence check algorithm. As a consequence, converged iterations can be detected independently within a Fortran module. Information about the simulation process (e.g., start of a new increment, execution of a next iteration or creation of a new crack segment and restart of a current increment) is “passed” to Abaqus by defining a user element with a very large label (to be called as a last finite element in an iteration). This element (not attached to the model analyzed) returns a very large force vector in iterations when the start of the next increment is not permitted or zero force vector in the opposite case.

The arc-length method has been used to control the simulation process. Generally, in arc-length methods, a system of balance equations in an iteration ***i*** can be written as:

(21) $$\begin{array}{c} K^{\left(i-1\right)}\delta u_{r}^{\left(i\right)}=r^{\left(i\right)}\\ K^{\left(i-1\right)}\delta u_{f}^{\left(i\right)}=f^{\left(i\right)} \end{array}$$

where K is the global stiffness matrix, r is the residuum force vector and f stands for a vector of external loads. Corrections $\delta u_{r}$ and $\delta u_{f}$ form the correction of total displacements:

(22) $$\delta u_{t}^{\left(i\right)}=\delta u_{r}^{\left(i\right)}+\delta \lambda^{\left(i\right)}\delta u_{f}^{\left(i\right)}$$

With the multiplier correction δλ. Abaqus Standard includes only one method based on arc-length control, namely modified Riks procedure. This approach is suitable in global buckling analysis, but it is not efficient in cases when deformations concentrate in small regions with elastic unloading in the remaining part. So-called indirect displacement control method is used here [61]. In order to implement it into Abaqus, some modifications are required. First the set containing all nodes of the model is copied and a new set is created with the same number of nodes and their original coordinates. The first and the second set of nodes store $u_{r}$ and $u_{f}$ displacements, respectively. The definition of a user element contains a subset of nodes from the first set following an analogous subset of nodes from the second set. The total number of nodes defining the element is doubled and the element stiffness matrix and element force vector is extended using the idea presented in Equation (21). Note that all displacements: $u_{r}$, $u_{f}$ and $u_{t}$ contain both standard and enriched displacement terms $a_{I}$ and $b_{I}$ respectively. Another user element with zero stiffness matrix and force vector is defined with nodes located at the ends of the gauge. It is responsible for modifying the value of the λ multiplier based on displacement values in nodes. Its label is manually set to one to ensure its call as the first element in the iteration.

Abaqus is not able to visualize user elements in Complete Abaqus Environment (CAE). Therefore, a third set of nodes is defined (again with the same original coordinates). Based on information from the original mesh, a set of built-in standard finite elements is created on these nodes. A zero stiffness (and stress) material is assigned to these elements. Information from user elements about strains and stresses is passed via module written in Fortran (Intel Company, Santa Clara, CA, USA) and next it is exported as state variables in these elements. In each node from the third set a one-node user element is created with the unit matrix as a stiffness matrix and a force vector with appropriate terms from the total displacement vector $u_{t}$. In that way global displacements can be visualized. However, this trick does not allow for presenting the crack pattern. It is achieved by creating Postscript files with deformed (and cracked) FE mesh in selected simulation times. Alternatively, each built-in standard finite element defined to visualize results, in which a crack occurs, is replaced with three or four built-in standard triangle elements. These elements are defined on standard nodes from the ‘master’ element and two additional pair of nodes located at the crack segment ends. It enables to visualize the growth of the crack during the simulation. However, this method requires some extra modifications of the input file and the re-execution of the simulation (information about the crack geometry is available after the completion of the job).

## 5. FE-Simulations

### 5.1. Input Data

The performance of all 18 beams are simulated, following the geometry data provided in Section 3. Steel blocks are also created with load/support points defined in the middle at the horizontal edge. Indirect displacement control method described in Section 4.5 is used to apply the load. The gauge length varies for the different beam’s sizes and shapes (between 12.7 mm and 162 mm) and it is taken directly from the experiment [32]. The ultimate elongation of the gauge is set to Δ = 0.3 mm. A requirement of execution of at least 1000 and 250 increments is imposed to complete the job in simulating unnotched and notched beams, respectively. The starting point for a discrete crack is defined manually in the middle of the horizontal edge at the top of the notch. The numerical calculations are carried out assuming plane stress conditions. Triangular constant strain finite elements are used. The FE mesh in the central region above the notch along the height is refined with the maximum length of a finite element side about 2 mm. The total number of finite elements is between 4981 and 11,660, depending on the beam geometry.

Elastic constants are the same as determined in the experiment. The Young’s modulus is taken as E = 41.24 GPa and the Poisson’s ratio is ν = 0.172 [32,41]. Note that a slightly different value of Young’s modulus has been assumed in [50]. They took E = 35.6 GPa based on the initial slope of the experimental load-displacement curve obtained from the largest unnotched specimen. The total fracture energy is fixed to $G_{F}\;$= 70 N/m (after [41]), although initially larger values $G_{F}\;$= 96.94 N/m and $G_{F}\;$= 110.09 N/m were reported [33]. Lorentz [43] assumed $G_{F}\;$= 75 N/m and Barbat et al. [45] took $G_{F}\;$= 90 N/m. In the calculations with the bilinear softening or the Bezier rational curve the traction at the kink point is always taken as $t_{k}\;$= 0.15$f_{t}$ (after [41]). The shear stiffness is taken as $K_{s}\;$= 0.0 N/m^3^. The companion calculations with other values of $K_{s}$ showed no difference in results. The remaining parameters: the tensile strength $f_{t}$, the initial fracture energy $G_{f}$, the weight w (for the Bezier rational curve) and the type of the softening curve vary thorough the simulations. The behavior of the steel loading plates is simulated by defining linear elastic constitutive law with the Young’s modulus $E_{s}$ = 200 GPa and the Poisson’s ratio $\nu_{s}\;$= 0.3. If not explicitly stated, no boundary layer reduction is applied.

### 5.2. Error Measures

In order to quantitatively estimate the quality of the simulation results, several error measures are used. The following relative error $Err_{0}$ defined as:

(23) $$Err_{0}=\frac{\sigma_{N}^{FEM}-\sigma_{N}^{Exp}}{\sigma_{N}^{Exp}}$$

is used to evaluate a single simulation. Here $\sigma_{N}^{FEM}$ is the nominal strength calculated from FE results as (using Equation (1)):

(24) $$\sigma_{N}^{FEM}=C_{f}\frac{3}{2}\cdot \frac{2.176P_{u}}{BD}$$

The whole set of N results (usually N = 18) is rated using mean percentage error $Err_{1}$:

(25) $$Err_{1}=\frac{1}{N}\overset{N}{\underset{i=1}{\sum }}Err_{0,i}$$

or mean absolute percentage error $Err_{2}$:

(26) $$Err_{2}=\frac{1}{N}\overset{N}{\underset{i=1}{\sum }}\left|Err_{0,i}\right|$$

### 5.3. Bilinear Softening

The choice of the values of the material parameters in softening to obtain the best fit is not an easy task. Havlásek et al. [50] calculated an error measure considering six force values for the huge beams (D = 500 mm) and peak loads for the remaining sizes. Lorentz [43] identified tensile parameters simulating medium (D = 93 mm) and large (D = 213 mm) beams with notch to depth ratios $\alpha_{0}\;$= 0.15 and $\alpha_{0}\;$= 0.30. Then he used these values to predict the behaviour of unnotched medium and large beams, and huge (D = 500 mm) unnotched and notched ($\alpha_{0\;}$= 0.15 and $\alpha_{0}\;$= 0.30) specimens. It is interesting to note that he did not simulate the performance of the small (D = 40 mm) beams at all. Grégoire et al. [35] calibrated their model parameters on the smaller beam sizes and they used them to simulate the behaviour of the largest specimens.

#### 5.3.1. Huge and Small Beams

**Figure 5:**
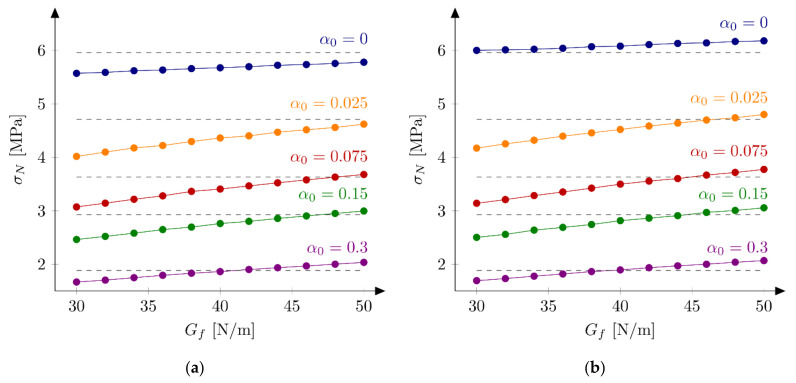
Nominal strengths $\sigma_{N}^{FEM}$ for the huge beam, different initial fracture energies and the tensile strength: (**a**) $f_{t}$ =4.8 MPa; (**b**) $f_{t}$ = 5.2 MPa.

**Figure 6:**
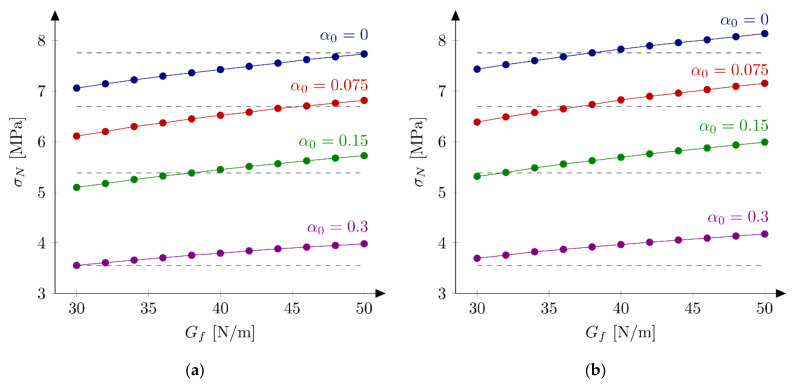
Nominal strengths $\sigma_{N}^{FEM}$ for the small beam, different initial fracture energies and the tensile strength: (**a**) $f_{t}\;$ = 4.8 MPa; (**b**) $f_{t}\;$ = 5.2 MPa.

First series of FE-calculations are performed using a bilinear softening law (Equation (10)). Here two “extreme” case are investigated. After some initial studies two values of the tensile strength are chosen for further calculations: $f_{t}\;$= 4.8 MPa and $f_{t}\;$= 5.2 MPa. A discrete set of initial fracture energies $G_{f}$ in the range of 30 to 50 N/m and an increment of 2 N/m is assumed. The remaining parameters are kept fixed with their initial values specified in Section 5.1. Figure 5 and Figure 6 present obtained nominal strengths $\sigma_{N}^{FEM}$ for the huge (D = 500 mm) and small (D = 40 mm) unnotched and notched specimens, respectively. In the simulations of the huge beams, the best results are obtained for the initial fracture energy $G_{f}\;$= 48 N/m ($Err_{1\;}$= 0.07% and $Err_{2}\;$= 6.20%) and $G_{f}\;$= 42 N/m ($Err_{1}\;$= −0.34%, $Err_{2}\;$= 2.72%) taking the tensile strength $f_{t}\;$= 4.8 MPa and $f_{t}\;$= 5.2 MPa, respectively. Taking the tensile strength $f_{t}\;$= 4.8 MPa, the larger notch to depth ratio $\alpha_{0}$ is assumed, the smaller initial fracture energy $G_{f}$ gives the best results. The same conclusion is true for the results with the tensile strength $f_{t}\;$= 5.2 MPa with an exception of the unnotched beams. It is interesting to remark that the sensitivity of the nominal strengths $\sigma_{N}^{FEM}$ with the respect to the initial fracture energy $G_{f}$ increases with increasing the notch to depth ratio $\alpha_{0}$, e.g., the error $Err_{0}$ is between 0.73% and 3.72% and between −10.13% and 9.76% for the notch to depth ratio $\alpha_{0}\;$= 0.0 and $\alpha_{0}\;$= 0.30, respectively (with the tensile strength $f_{t}\;$= 5.2 MPa).

Calculations of the small beams give the best results for the initial fracture energy $G_{f}\;$= 40 N/m ($Err_{1}\;$= 0.32% and $Err_{2}\;$= 6.91%) and $G_{f}\;$= 32 N/m ($Err_{1}\;$= −0.03%, $Err_{2}\;$= 5.84%) taking the tensile strength $f_{t}\;$= 4.8 MPa and $f_{t}\;$= 5.2 MPa, respectively. Both simulation sets confirm the previous observation of decreasing the “best” initial fracture energy $G_{f}$ with increasing the notch to depth ratio $\alpha_{0}$. The sensitivity of calculated nominal strengths for the small beams with the respect to notch to depth ratio $\alpha_{0}$ is smaller comparing with results for the huge beams. The error $Err_{0}$ is between −4.17% and 4.90% and between 4.08% and 17.56% for the notch to depth ratio $\alpha_{0}\;$= 0.0 and $\alpha_{0}\;$= 0.30, respectively (with the tensile strength $f_{t}\;$= 5.2 MPa). Graphically this fact may be seen by comparing line inclinations at Figure 5 and Figure 6 for different notch to depth ratios $\alpha_{0}$. Analogous parametric studies for large (D = 215 mm) and medium (D = 93 mm) beams confirm the observation the “best” initial fracture energy $G_{f}$ decreased with decreasing the specimen’s size (e.g., the initial fracture energy with $f_{t}\;$= 5.2 MPa is found to be $G_{f}\;$= 38 N/m for both large and medium beams).

#### 5.3.2. All Beam Geometries

The definition of objective quality measures allows for choosing the best parameters set. Of course, such analysis should be performed with the restriction of the stochastic nature of the experimental results. Therefore, the average values of maximum forces obtained experimentally (which serve to asset simulation results) due to finite low number of realizations (specimens tested) can also introduce some errors in the analysis. In experiments by Hoover et al. [32] coefficients of variation were approximately about 5% (they can be interpreted as results scatter). However, in order to limit the number of simulations executed, no stochastic analysis will be performed here, and averaged values of experimental maximum forces will be treated as ‘perfect’ ones.

**Figure 7:**
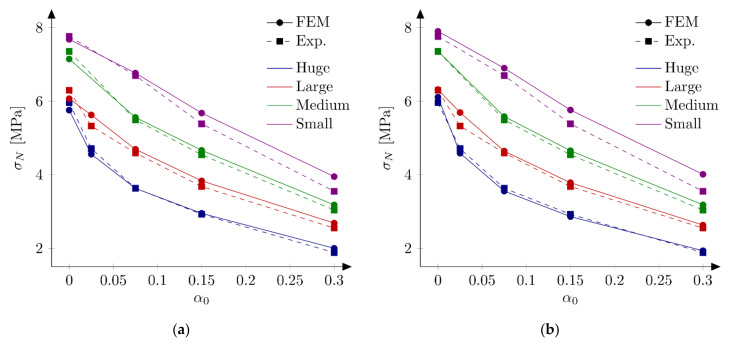

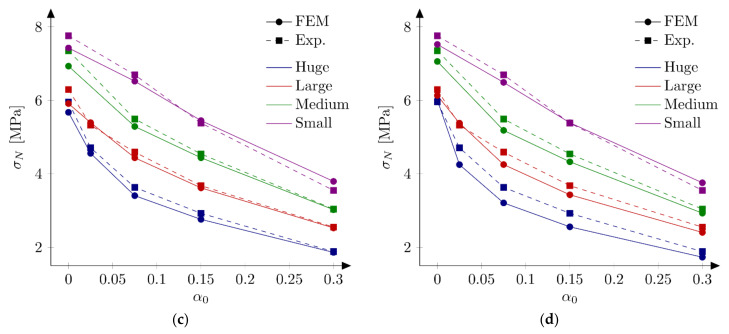
Nominal and experimental strengths with bilinear softening law and: (**a**) set S1: $f_{t}\;$ = 4.8 MPa and $G_{f}\;$ = 48 N/m; (**b**) set S2: $f_{t}\;$ = 5.2 MPa and $G_{f}\;$ = 42 N/m; (**c**) set S3: $f_{t}\;$ = 4.8 MPa and $G_{f}\;$ = 40 N/m; (**d**) set S4: $f_{t}\;$ = 5.2 MPa and $G_{f}\;$ = 32 N/m.

On the basis of the results from the Section 5.3.1., four sets of parameters are chosen to perform calculations with all 18 beams: set S1: $f_{t}\;$= 4.8 MPa and $G_{f}\;$= 48 N/m, set S2: $f_{t}\;$= 5.2 MPa and $G_{f}\;$= 42 N/m (both from simulations of the huge beams), set S3: $f_{t}\;$= 4.8 MPa and $G_{f}\;$= 40 N/m, and set S4: $f_{t}\;$= 5.2 MPa and $G_{f}\;$= 32 N/m (both from simulations of the small beams). Table 2 presents calculated nominal strengths obtained with sets S1–S4. Graphical comparison of experimental and numerical nominal strengths is depicted at Figure 7. The following errors are obtained: set S1: $Err_{1}\;$= 2.02% and $Err_{2}\;$= 3.59%, set S2: $Err_{1}\;$= 2.57% and $Err_{2}\;$= 3.34%, set S3: $Err_{1}\;$= −2.60% and $Err_{2}\;$= 3.65%, and set S4: $Err_{1}\;$= −4.47% and $Err_{2}\;$= 5.36%. In the calculations with sets S1 and S2 the largest errors are obtained for the small beam and the notch to depth ratio $\alpha_{0}$ = 0.3 ($Err_{0}\;$= 11.22% and $Err_{0}\;$= 12.97% for set S1 and S2, respectively). The absolute values of the error *Err*_0_ do not exceed 7% for the remaining geometries. Generally nominal strengths are overestimated for the small beams, while the agreement with the experimental outcomes is very good for the other specimens (especially for the set S2). On the contrary, results with sets S3 and S4 generally underestimate experimental peak loads for larger beams (especially when parameters set S4 is assumed).

The obtained results reveal some problems in determining optimum material parameters in concrete. Sets S1, S2 and S3 give similar errors $Err_{2}$. Sets S1 and S2 overestimate the peak loads, while set S3 underestimate it in average, but the absolute values of the errors $Err_{1}$ are similar. Only calculations with set S4 produce larger both errors. Analysis of nominal strengths obtained from FE-calculations reported in [41] return errors $Err_{1}\;$= −3.76% and $Err_{2}\;$= 4.43%. These values are larger than the errors obtained with sets S1, S2 and S3, but they are smaller than errors from results with the set S4. Taking material parameters from [41] and using the approach described here (XFEM) much larger errors are achieved: $Err_{1}\;$= −7.29% and $Err_{2}\;$= 7.52%. The second comment should be made about the value of the tensile strength $f_{t}$. The values between 4.8 MPa and 5.2 MPa are taken here, while Hoover and Bažant [41] assumed the tensile strength equal to $f_{t}\;$= 3.92 MPa. On the other hand, Havlásek et al. [50] found the optimum uniaxial tensile strength as $f_{t}\;$= 4.984 MPa. Lorentz [43] used a similar value, namely $f_{t}\;$= 5.0 MPa. Note also that this high value of the tensile strength corresponds nicely with the nominal strength for very large structures $f_{r,\infty }\;$= 5.27 MPa determined in [33].

A comment should be made about application of XFEM to simulate unnotched beams under bending with only one crack. The exact solution should consider a region in the middle at the bottom edge of the beam with several initial cracks. At the beginning a region of diffuse damage is formed. Upon increasing the loading force, cracks develop, but one crack dominates (in the case of the problem analysed here with the axis of symmetry it would be crack located along this axis). Planas et al. [62] showed numerically that in unnotched beams under bending, several cracks start to develop but only one crack dominates at the peak. Moreover, obtained errors with calculations of unnotched beams with only one crack defined are similar to values obtained for beams with $\alpha_{0}\;$= 0.025 and they confirm general trends observed in Section 5.3.1. Even two different size effect laws can be postulated to describe unnotched (Type I) and notched (Type II) beams, XFEM with this simplified approach is able to capture numerically both these phenomena. Therefore, this simplification (one crack instead of a bundle of cracks) is justified. The same simplification was made by Fend and Wu [44]. In the simulations of the unnotched beams they also assumed only one crack starting from the midpoint of the bottom edge of a beam.

**Figure 8:**
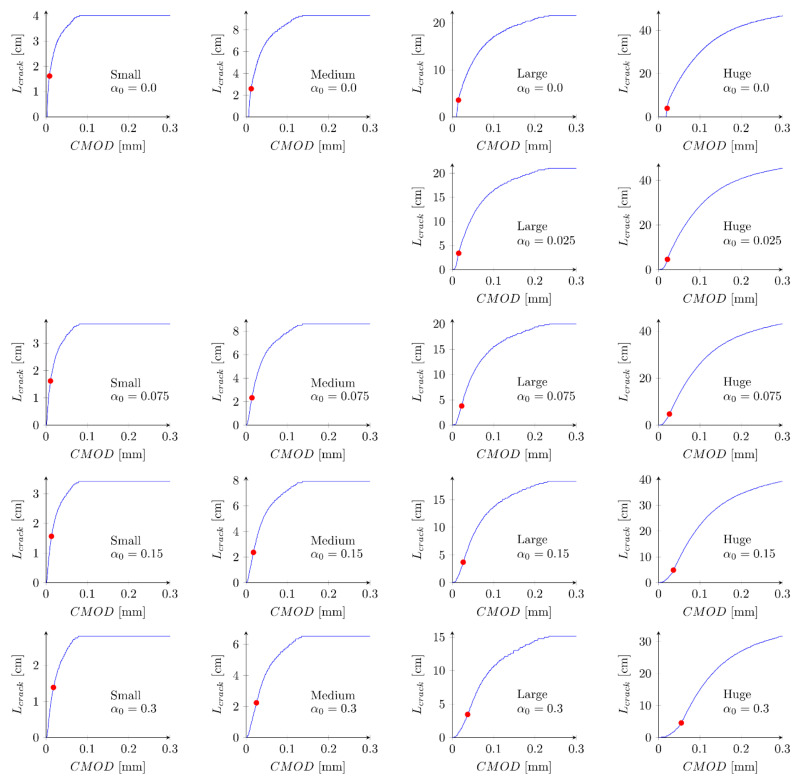
Evolution of crack lengths versus crack mouth opening displacement for set S1: $f_{t}\;$= 4.8 MPa and $G_{f}$ = 48 N/m.

Figure 8 presents evolution of the cracks’ lengths versus the crack-mouth open displacements for all beams and parameters set S1. Red points indicate the moment when the maximum force is obtained. The crack length $L_{crack}$ at the peak is in range 1.4–1.6 cm ($L_{crack}/D\;$= 0.35–0.40), 2.3–2.6 cm ($L_{crack}/D\;$= 0.25–0.28), 3.4–3.8 cm ($L_{crack}/D\;$= 0.16–0.18) and 3.9–4.9 cm ($L_{crack}/D\;$= 0.08–0.10) for small, medium, large and huge beams, respectively. The larger the beam is assumed, the smaller the relative crack length is obtained [63]. For small beams a crack is always fully developed (a large horizontal plateau) while for the huge beams a crack is still to propagate (no horizontal plateau). In general, at the peak a crack is far away from being fully formed.

#### 5.3.3. Boundary Layer

**Figure 9:**
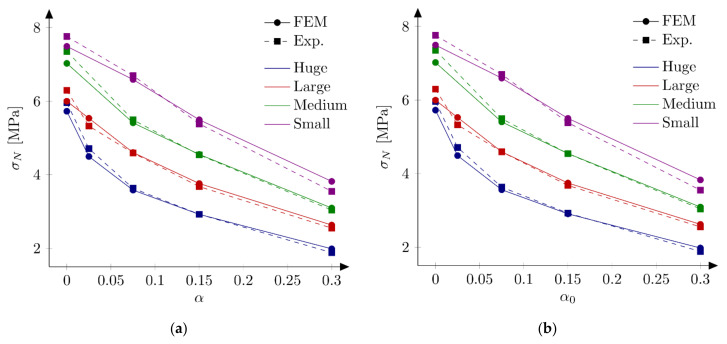

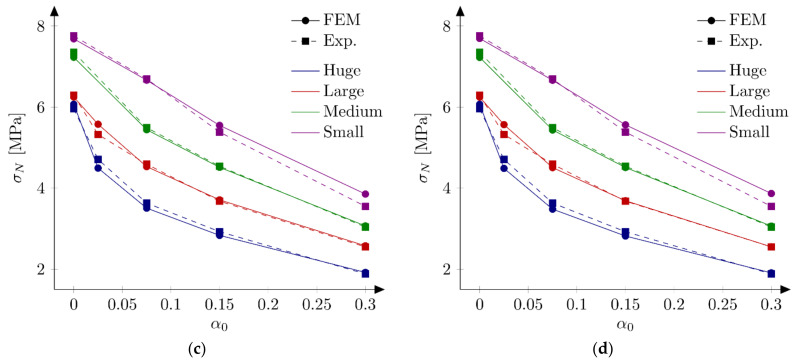
Nominal and experimental strengths with bilinear softening law and: (**a**) set S5: $f_{t}$ = 4.8 MPa, $G_{f}\;$ = 48 N/m, b  = 0.5 cm, $\beta_{0}\;$ = 0.7; (**b**) set S6: $f_{t}\;$ = 4.8 MPa,$\;G_{f}$ = 48 N/m, b  = 1.0 cm, $\beta_{0}\;$ = 0.8; (**c**) set S7: $f_{t}\;$ = 5.2 MPa, $G_{f}\;$ = 42 N/m, b  = 0.5 cm, $\beta_{0}\;$ = 0.7; (**d**) set S8: $f_{t}$ = 5.2 MPa, $G_{f}\;$ = 42 N/m, b  = 1.0 cm, $\beta_{0}\;$ = 0.8.

**Figure 10:**
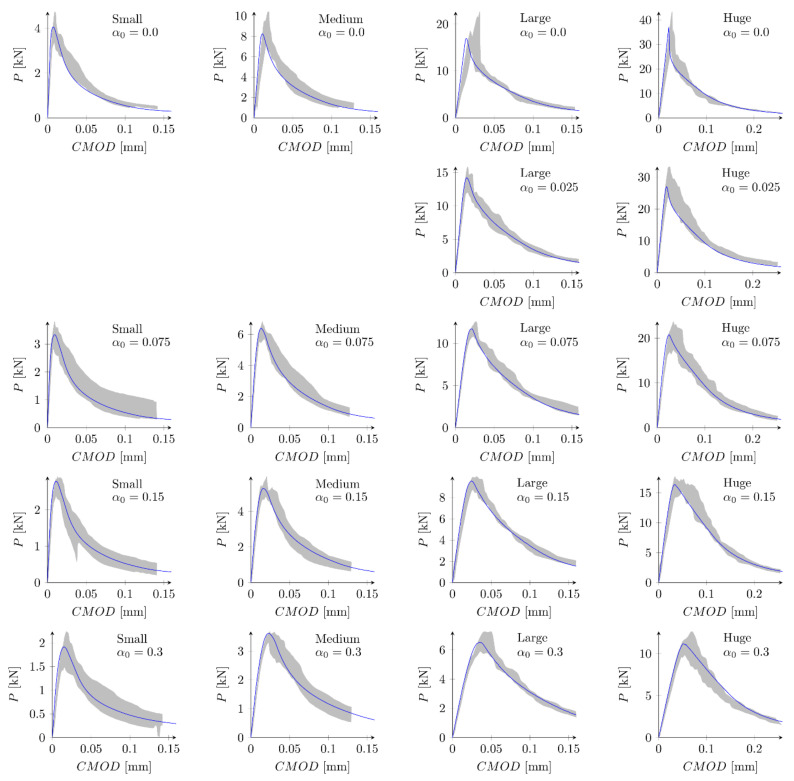
Experimental and numerical force—crack mouth opening displacement curves for bilinear softening and set S7: $f_{t}\;$= 5.2 MPa, $G_{f\;}\;$ = 42 N/m, b  = 0.5 cm, $\beta_{0}\;$ = 0.7.

In the next phase, the influence of the boundary layer is examined. Material parameters S1 and S2 are adopted. For each parameters set two values of the boundary layer thickness are analyzed: *b* = 0.5 cm and *b* = 1.0 cm. Nine discrete values of the reduction coefficient at the edge $\beta_{0}$ in the range of 0.1 to 0.9 and the increment of 0.1 are assumed. Based on simulations of all geometries and the analysis of errors $Err_{1}$ and $Err_{2}$ the best values of the coefficient $\beta_{0}$ are determined. Finally four new parameter sets are defined: S5: $f_{t}\;$= 4.8 MPa, $G_{f}\;$= 48 N/m, b = 0.5 cm and $\beta_{0}\;$= 0.7, S6: $f_{t}\;$= 4.8 MPa, $G_{f\;}$= 48 N/m, b = 1.0 cm and $\beta_{0}\;$= 0.8, S7: $f_{t}\;$= 5.2 MPa, $G_{f}\;$= 42 N/m, b = 0.5 cm, $\beta_{0}\;$= 0.7 and S8: $f_{t}\;$= 5.2 MPa, $G_{f\;}$= 42 N/m, b = 1.0 cm, $\beta_{0}\;$= 0.8. In above sets almost the identical and relatively high values of the coefficients $\beta_{0}$ are assumed for different values of the boundary layer thickness. Figure 9 shows calculated nominal strengths compared with experimental values and calculated nominal strengths are listed in Table 3. Set of force—crack mouth opening displacement (CMOD) curves for all 18 beams is presented at Figure 10 (set S7), with grey areas between experimental extreme curves (here numerical force values are not corrected with the factor $C_{f}$). It can be seen all numerical results fit into experimental limits. The error measures are equal to: $Err_{1}\;$= 0.09% and $Err_{2}\;$= 2.92%, $Err_{1}\;$= −0.12% and $Err_{2}\;$= 2.90%, $Err_{1}\;$= 0.30% and $Err_{2}\;$= 2.27%, and $Err_{1}\;$= 0.08% and $Err_{2\;}$= 2.28% for the set S5, S6, S7 and S8, respectively. In all cases the largest error $Err_{0}$ is obtained for the small beam with the notch to depth ratio $\alpha_{0}\;$= 0.3 and it is about 9%. The errors $Err_{0}$ from the remaining beams do not exceeded 5.5% (its absolute values). Comparing with simulations S1–S4 the presence of the boundary layer decreases the error measures, especially for the sets with the tensile strength equal to $f_{t}\;$= 5.2 MPa (set S2 versus sets S7 and S8). On the other hand, input parameters (S1 and S2) already produce relatively small errors. By further parametric studies even better parameters can be found. For instance FE-calculations with the following parameters (set S9): $f_{t}\;$= 5.0 MPa and $G_{f}\;$= 48 N/m give the following errors: $Err_{1}\;$= 0.67% and $Err_{2}\;$= 2.69%, comparable with errors obtained with sets with declared boundary layer. Therefore, the definition of the boundary cannot be treated as the significant improvement of the results. Simulation results do not allow also for unique identification of the boundary layer thickness.

#### 5.3.4. Notched Beams

**Figure 11:**
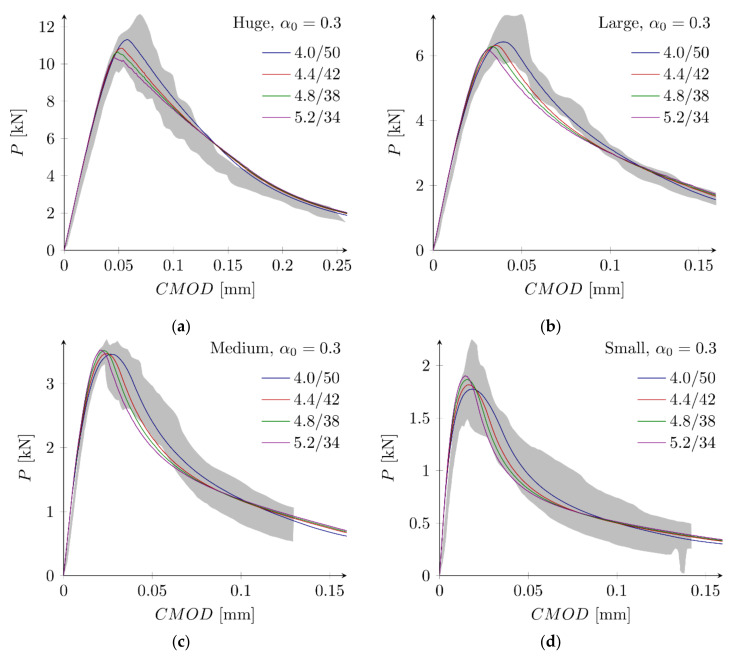
Force–displacement curves for notched ($\alpha_{0}\;$= 0.3) beams and sets N1–N4: (**a**) huge beam; (**b**) large beam; (**c**) medium beam; (**d**) small beam (the first number in a label is the tensile strength in MPa, the second number is the initial fracture energy in N/m).

As an alternative approach, notched beams with the notch to depth ratio equal to $\alpha_{0}\;$= 0.3 are used to determine the fracture parameters. Based upon parametric studies, the following sets are defined: set N1 with $f_{t}\;$= 4.0 MPa, $G_{f}\;$= 50 N/m, set N2 with $f_{t}\;$= 4.4 MPa, $G_{f}\;$= 42 N/m, set N3 with $f_{t}\;$= 4.8 MPa, $G_{f}\;$= 38 N/m, and set N4 with $f_{t}\;$= 5.2 MPa, $G_{f}\;$= 34 N/m. They give the following values of the error measures (calculated only from four beam sizes with $\alpha_{0}\;$= 0.3): $Err_{1}\;$= −0.02% and $Err_{2}\;$= 1.90%, $Err_{1}$ = −0.89% and $Err_{2}\;$= 2.30%, $Err_{1}\;$= 0.04% and $Err_{2}\;$= 2.85%, and $Err_{1}\;$=−0.96% and $Err_{2}\;$= 4.81%, with the set N1, N2, N3 and N4, respectively. Figure 11 shows obtained force–displacement curves. In general, comparable agreement with experimental outcomes is achieved for all parameter sets. For the smallest beam, the maximum load is obtained with the set N4 and the minimum load with the set N1, while for the largest beam the opposite case occurs. Force–displacement diagrams for the small beam suggest also that the assumed here total fracture energy $G_{F}$ = 70 N/m is too small. This observation is consistent with the larger value of the total fracture energy ($G_{F}\;$= 96.94 N/m) calculated by Hoover and Bažant [14] on the basis of experimental results. On the other hand, simulations of all beam sizes with sets N1–N4 generally underestimate experimental results. They return errors $Err_{1}\;$= −6.09% and $Err_{2}\;$= 6.51%, $Err_{1}\;$= −5.53% and $Err_{2}\;$= 5.85%, $Err_{1}\;$= −3.53% and $Err_{2}\;$= 4.19%, and $Err_{1}\;$= −2.91% and $Err_{2}\;$= 4.32%, for the set N1, N2, N3 and N4, respectively.

### 5.4. Exponential Softening

**Figure 12:**
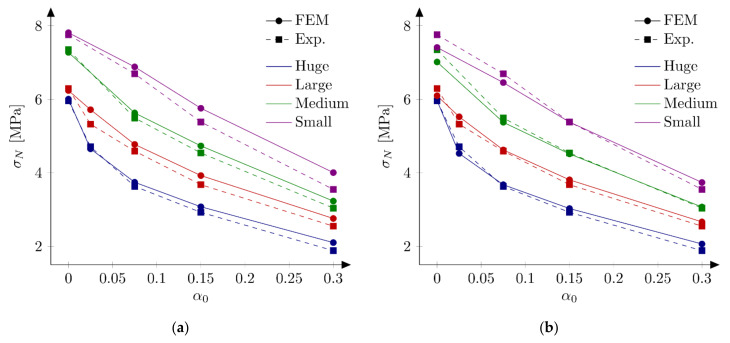
Nominal and experimental strengths with exponential softening law and: (**a**) set S10: $f_{t}\;$= 5.2 MPa and $G_{f}\;$ = 35 N/m; (**b**) set S11: $f_{t}\;$ = 5.2 MPa, $G_{f}\;$ = 35 N/m, b = 1 cm, $\beta_{0}\;$ = 0.6.

**Figure 13:**
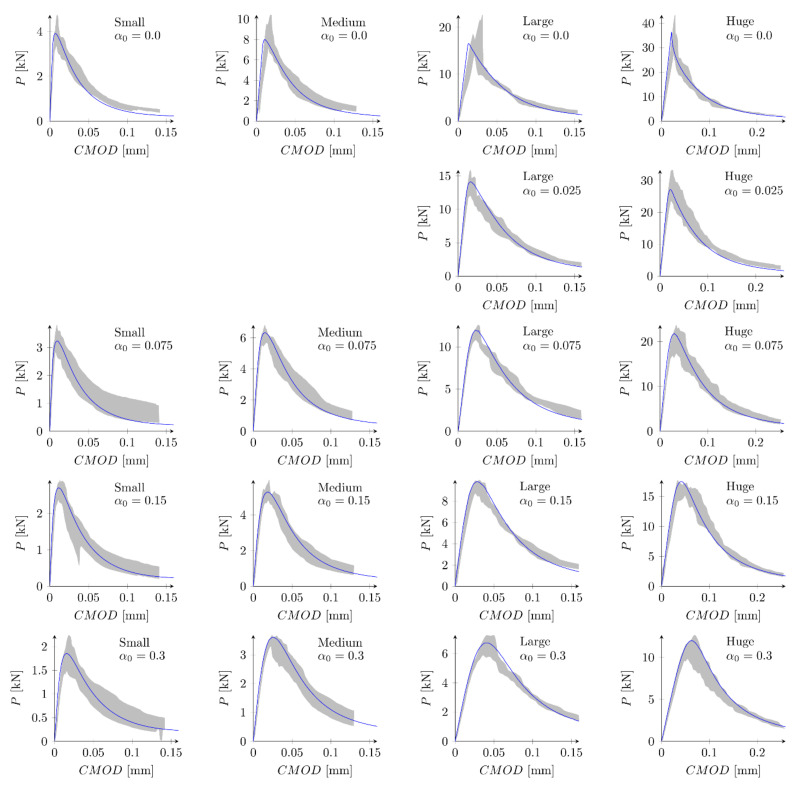
Experimental and numerical force-crack mouth opening displacement curves for exponential softening and set S11: $f_{t}$= 5.2 MPa, $G_{f}\;$ = 35 N/m, b  = 1 cm, $\beta_{0}\;$ = 0.6.

So far, only a bilinear softening law has been used. The same curve was used by Hoover and Bažant [41] who postulated that a bilinear shape of the softening curve is a fundamental property of concrete. They also stated that no linear nor exponential functions in softening allowed for fitting numerical results with experiments. Despite this information Havlásek et al. [50] and Grégoire et al. [35] used exponential relationships in their simulations with isotropic damage models with non-local softening (to be more precise different formulas involving exponent function were used in both papers). Lorentz [43] also proposed a formula containing an exponential function to describe the post-peak behavior. In order to clarify this issue, FE-calculations with the exponential softening law (Equation (12)) are performed. The initial fracture energy is equal to $G_{f}\;$= 35 N/m (50% of the total fracture energy). The tensile strength is assumed as $f_{t}\;$= 5.2 MPa (set S10). Obtained nominal strengths are presented at Figure 12a and in Table 4. The error measures are equal to $Err_{1}\;$= 4.46% and $Err_{2}\;$= 4.80%. They are larger than obtained with sets S1–S3, but comparable with errors from original simulations (Hoover and Bažant [41]). The application of the boundary layer model (set S11 with $f_{t}\;$= 5.2 MPa, $G_{f}\;$= 35 N/m, b = 1 cm and $\beta_{0}\;$= 0.6) significantly improves the results. The error measures are equal to $Err_{1}\;$= 0.61% and $Err_{2}$= 3.10%. Comparison between numerical and experimental nominal strengths is made in Figure 12b. Family of force—CMOD diagrams is shown at Figure 13. Generally, all numerical curves fall into experimental limits. Again, the application of the boundary layer is not necessary if better parameters are found. Taking $f_{t}\;$= 4.8 MPa and $G_{f}\;$= 35 N/m the errors are calculated as $Err_{1}\;$= 0.20% and $Err_{2\;}\;$= 3.65% (set S12).

### 5.5. Bezier Rational Curve

**Figure 14:**
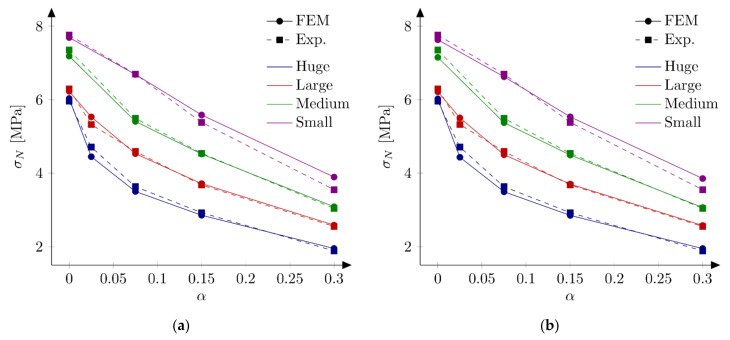
Nominal and experimental strengths with rational Bezier softening law and: (**a**) set S13: $f_{t}\;$= 5.2 MPa, $G_{f}\;$ = 35 N/m, w  = 4; (**b**) set S14: $f_{t}\;$ = 5.2 MPa, $G_{f}\;$ = 35 N/m, w  = 4, b = 1 cm, $\beta_{0}\;$ = 0.9.

**Figure 15:**
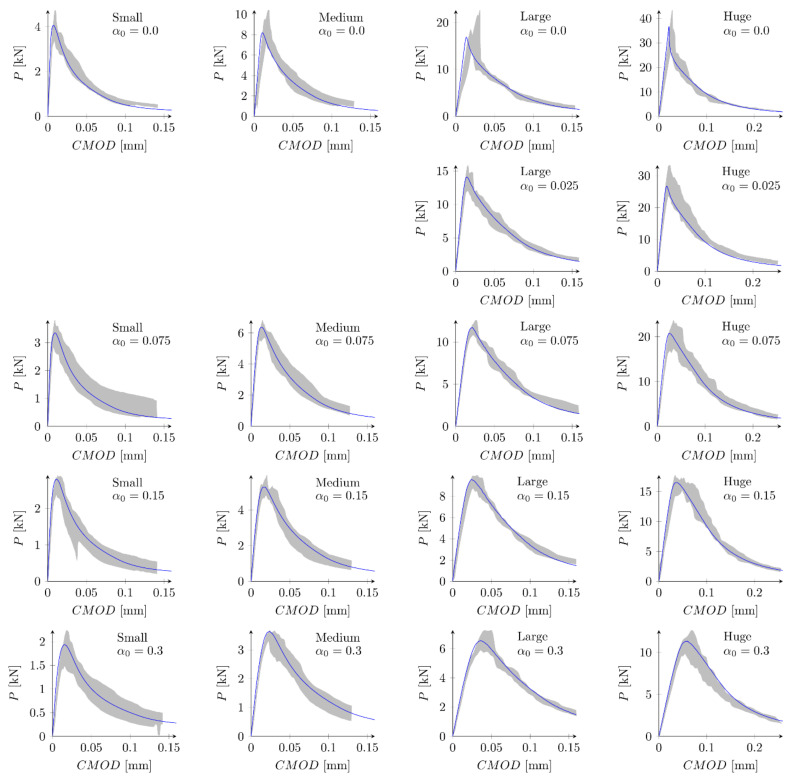
Experimental and numerical force—crack mouth opening displacement curves for rational Bezier softening and set S13: $f_{t}\;$= 5.2 MPa, $G_{f}\;$ = 35 N/m, w  = 4.

Finally, simulations with the softening law based on Bezier rational curve (Equation (13)) are executed. The set S13 is defined with the tensile strength $f_{t}\;$= 5.2 MPa, initial fracture energy $G_{f}\;$= 35 N/m and the weight w = 4 (parameters chosen on some initial simulations). The fracture energy $G_{B}$ is equal 59.52 N/m. Obtained numerical nominal strengths are shown at Figure 14 and listed in Table 4, while force-CMOD diagrams are depicted in Figure 15. The calculated error measures are $Err_{1}\;$= 0.38% and $Err_{2}\;$= 2.51% (values comparable with improved parameter sets S4–S8). All numerical curves fit the experimental limits. What is interesting is the use of the boundary layer method (set S14: $f_{t}\;$= 5.2 MPa, $G_{f}\;$= 35 N/m, w = 4, b = 1 cm, $\beta_{0}\;$= 0.9) decreases the error $Err_{1}\;$= −0.13%, but it slightly increases the error, $Err_{2\;}$= 2.55%.

## 6. Conclusions

Numerical simulations of unnotched and notched geometrically similar concrete beams of different sizes and different notch to depth ratios have been presented. Obtained results have been compared with experimental data [32]. Two error measures have been defined and used to quantitatively assess calculated maximum forces. The influence of the softening law has been investigated. Three alternatives have been examined: bilinear, exponential and ration Bezier curves. All analysed softening curves turn out to be equivalently good, they give results with comparable error measures consistent with experiments. This conclusion contradicts the hypothesis of the supremacy of the bilinear definition postulated by Hoover and Bažant [41]. At the same time, the use of different softening laws results in different values of best initial fracture energies. This fact reveals some limitations of the initial fracture energy definition when a nonlinear relationship is assumed instead of segmentally linear function. The linear reduction of the initial and total fracture energies in the boundary layer did not significantly improve the results. The assumed value of the total fracture energy $G_{F}\;$= 70 N/m based on analysis of the experimental curves performed by Hoover and Bažant [33], was correct. Obtained force–displacement diagrams fitted within experimental curves.

Simulations with the cohesive crack model were the first step. The ongoing research aim is to define an equivalence of initial fracture energy definition for different softening laws, especially for non-linear relationships. It will lead to a more unique definition of this quantity and to a better understanding of the fracture process.

## Figures and Tables

**Table 1:** Experimental results: nominal strengths $\sigma_{N}^{Exp}$ [32], correction factors $C_{f}$ and averaged maximum forces $P_{u}^{Exp}$ (bold number–refer to explanation given in text).

D [mm]	$\alpha_{0}$	$\sigma_{N}^{Exp}[\mathrm{MPa}]$	$C_{f}$	$P_{u}^{Exp}[\mathrm{kN}]$
40	0	7.756	0.927	4.10
40	0.075	6.694	0.978	3.35
40	0.15	5.383	0.975	2.71
40	0.30	3.550	0.986	1.76
93	0	7.350	0.880	9.52
93	0.075	5.492	0.968	6.47
93	0.15	4.541	0.975	5.31
93	0.30	3.041	0.967	3.58
215	0	6.295	0.972	17.07
215	0.025	5.323	1.032	13.59
215	0.075	4.591	1.018	11.88
215	0.15	3.678	1.023	9.47
215	0.30	2.551	1.042	6.45
500	0	5.956	1.009	36.18
500	0.025	4.710	1.021	28.27
500	0.075	3.632	1.034	21.53
500	0.15	2.926	1.060	16.91
500	0.30	1.884	1.053	10.97

**Table 2:** Calculated nominal strengths $\sigma_{N}^{FEM}$ for bilinear softening curves and sets S1–S4.

D [mm]	$\alpha_{0}$	$\sigma_{N}^{FEM}[\mathrm{MPa}]$			
		S1	S2	S3	S4
40	0	7.678	7.896	7.425	7.522
40	0.075	6.762	6.896	6.521	6.489
40	0.15	5.676	5.759	5.449	5.391
40	0.30	3.948	4.011	3.795	3.757
93	0	7.145	7.357	6.931	7.058
93	0.075	5.558	5.580	5.289	5.183
93	0.15	4.663	4.654	4.437	4.328
93	0.30	3.180	3.181	3.025	2.930
215	0	6.067	6.314	5.912	6.133
215	0.025	5.625	5.691	5.394	5.380
215	0.075	4.691	4.642	4.437	4.253
215	0.15	3.833	3.784	3.618	3.430
215	0.30	2.686	2.632	2.528	2.406
500	0	5.755	6.107	5.674	6.010
500	0.025	4.558	4.585	4.361	4.251
500	0.075	3.629	3.555	3.406	3.209
500	0.15	2.950	2.862	2.761	2.558
500	0.30	2.001	1.935	1.862	1.732

**Table 3:** Calculated nominal strengths $\sigma_{N}^{FEM}$ for bilinear softening curves and sets S5–S9.

D [mm]	$\alpha_{0}$	$\sigma_{N}^{FEM}[\mathrm{MPa}]$				
		S5	S6	S7	S8	S9
40	0	7.488	7.491	7.684	7.694	7.690
40	0.075	6.587	6.593	6.664	6.662	6.744
40	0.15	5.494	5.500	5.548	5.562	5.638
40	0.30	3.820	3.828	3.854	3.868	3.928
93	0	7.026	7.018	7.229	7.225	7.171
93	0.075	5.411	5.403	5.441	5.435	5.483
93	0.15	4.553	4.545	4.514	4.510	4.579
93	0.30	3.096	3.091	3.068	3.064	3.130
215	0	5.998	5.994	6.248	6.246	6.138
215	0.025	5.535	5.525	5.576	5.567	5.571
215	0.075	4.604	4.591	4.531	4.507	4.575
215	0.15	3.762	3.743	3.711	3.689	3.753
215	0.30	2.634	2.621	2.577	2.553	2.604
500	0	5.725	5.723	6.070	6.068	5.904
500	0.025	4.496	4.488	4.495	4.488	4.492
500	0.075	3.583	3.561	3.507	3.482	3.512
500	0.15	2.924	2.906	2.835	2.819	2.834
500	0.30	1.988	1.978	1.921	1.910	1.913

**Table 4:** Calculated nominal strengths $\sigma_{N}^{FEM}$ for exponential (sets S10–S12) and rational Bezier (sets S13–S14) softening curves.

D [mm]	$\alpha_{0}$	$\sigma_{N}^{FEM}[\mathrm{MPa}]$				
		S10	S11	S12	S13	S14
40	0	7.811	7.410	7.425	7.688	7.629
40	0.075	6.882	6.455	6.551	6.686	6.623
40	0.15	5.757	5.381	5.504	5.584	5.532
40	0.30	4.007	3.739	3.828	3.894	3.854
93	0	7.276	7.015	6.921	7.184	7.150
93	0.075	5.629	5.375	5.396	5.408	5.373
93	0.15	4.733	4.515	4.545	4.521	4.493
93	0.30	3.232	3.071	3.107	3.087	3.065
215	0	6.239	6.098	5.883	6.227	6.215
215	0.025	5.718	5.525	5.469	5.530	5.504
215	0.075	4.774	4.623	4.604	4.531	4.502
215	0.15	3.926	3.810	3.797	3.715	3.703
215	0.30	2.759	2.666	2.667	2.586	2.577
500	0	6.004	5.965	5.647	6.031	6.020
500	0.025	4.654	4.527	4.457	4.448	4.433
500	0.075	3.744	3.677	3.633	3.502	3.494
500	0.15	3.077	3.029	3.004	2.855	2.851
500	0.30	2.103	2.066	2.051	1.951	1.949

## Data Availability

Not applicable.

## References

[1] Pivonka P., Ožbolt J., Lackner R., Mang H.A. (2004). Comparative studies of 3D-constitutive models for concrete: Application to mixed-mode fracture. Int. J. Numer. Methods Eng..

[2] Marzec I., Tejchman J., Winnicki A. (2015). Computational simulations of concrete behaviour under dynamic conditions using elasto-visco-plastic model with non-local softening. Comput. Concr..

[3] Manzoli O.L., Oliver J., Diaz G., Huespe A.E. (2008). Three-dimensional analysis of reinforced concrete members via embedded discontinuity finite elements. IBRACON Struct. Mater. J..

[4] Skarżyński Ł., Marzec I., Tejchman J. (2017). Experiments and numerical analyses for composite RC-EPS slabs. Comput. Concr..

[5] Marzec I., Tejchman J., Mróz A. (2019). Numerical analysis of size effect in RC beams scaled along height or length using elasto-plastic-damage model enhanced by non-local softening. Finite Elem. Anal. Des..

[6] Skarżyński Ł., Marzec I., Drąg K., Tejchman J. (2020). Numerical analyses of novel prefabricated structural wall panels in residential buildings based on laboratory tests in scale 1:1. Eur. J. Environ. Civ. Eng..

[7] Grassl P., Jirásek M. (2006). Damage-plastic model for concrete failure. Int. J. Solids Struct..

[8] Mazars J., Hamon F., Grange S. (2015). A new 3d damage model for concrete under monotonic, cyclic and dynamic load. Mater. Struct..

[9] Wang X., Zhang M., Jivkov A.P. (2016). Computational technology for analysis of 3D meso-structure effects on damage and failure of concrete. Int. J. Solids Struct..

[10] Trawiński W., Tejchman J., Bobiński J. (2018). A three-dimensional meso-scale modelling of concrete fracture, based on cohesive elements and X-ray μCT images. Eng. Fract. Mech..

[11] Wells G.N., Sluys L.J. (2001). A new method for modelling cohesive cracks using finite elements. Int. J. Numer. Methods Eng..

[12] Unger J.F., Eckardt S., Könke C. (2007). Modelling of cohesive crack growth in concrete structures with the extended finite element method. Comput. Methods Appl. Mech. Eng..

[13] Im S., Ban H., Kim Y.-R. (2014). Characterization of mode-I and mode-II fracture properties of fine aggregate matrix using a semicircular specimen geometry. Constr. Build. Mater..

[14] Dong Z., Gong X., Zhao L., Zhang L. (2014). Mesostructural damage simulation of asphalt mixture using microscopic interface contact models. Constr. Build. Mater..

[15] Wang X., Li K., Zhong Y., Xu Q., Li C. (2018). XFEM simulation of reflective crack in asphalt pavement structure under cyclic temperature. Constr. Build. Mater..

[16] Haeri H., Sarfarazi V., Ebneabbasi P., Nazari Maram A., Shahbazian A., Fatehi Marji M., Mohamadi A.R. (2020). XFEM and experimental simulation of failure mechanism of non-persistent joints in mortar under compression. Constr. Build. Mater..

[17] Perego U., Comi C., Mariani S. (2007). An extended FE strategy for transition from continuum damage to mode I cohesive crack propagation. Int. J. Numer. Anal. Methods Geomech..

[18] Bobiński J., Tejchman J. (2016). A coupled constitutive model for fracture in plain concrete based on continuum theory with non-local softening and eXtended Finite Element Method. Finite Elem. Anal. Des..

[19] Petersson P.E. (1981). Crack Growth and Development of Fracture Zones in Plain Concrete and Similar Materials.

[20] Wittmann F.H., Rokugo K., Bruhwiler E., Mihashi H., Simopnin P. (1988). Fracture energy and strain softening of concrete as determined by compact tension specimens. Mater. Struct..

[21] CEB-90 (1991). Final Draft CEB-FIP Mode Code 1990.

[22] Bažant Z.P. (2002). Concrete fracture models: Testing and practice. Eng. Fract. Mech..

[23] Park K., Paulino G.H., Roesler J.R. (2008). Determination of the kink point in the bilinear softening model for concrete. Eng. Fract. Mech..

[24] Gopalaratnam V.S., Shah S.P. (1985). Softening response of plain concrete in direct tension. ACI J. Proced..

[25] Reinhardt H.W., Cornelissen H.A.W., Hordijk D.A. (1986). Tensile tests and failure analysis of concrete. J. Struct. Eng..

[26] Chen H.H., Su R.K.L. (2013). Tension softening curves of plain concrete. Constr. Build. Mater..

[27] Tang Y., Chen H. (2019). Characterizations on fracture process zone of plain concrete. J. Civ. Eng. Manag..

[28] Kumar S., Barai S.V. (2009). Effect of softening function on the cohesive crack fracture parameters of concrete CT specimen. Sadhana.

[29] Dong W., Wu Z., Zhou X. (2013). Calculating crack extension resistance of concrete based on a new crack propagation criterion. Constr. Build. Mater..

[30] Carpinteri A., Chiaia B., Cornetti P. (2003). On the mechanics of quasi-brittle materials with a fractal microstructure. Eng. Fract. Mech..

[31] Wang L., Zeng X., Yang H., Lv X., Guo F., Shi Y., Hanif A. (2021). Investigation and application of fractal theory in cement-based materials: A review. Fractal Fract..

[32] Hoover C.G., Bažant Z.P., Vorel J., Wendner R., Hubler M.H. (2013). Comprehensive concrete fracture tests: Description and results. Eng. Fract. Mech..

[33] Hoover C.G., Bažant Z.P. (2013). Comprehensive concrete fracture tests: Size effects of types 1 & 2, crack length effect and postpeak. Eng. Fract. Mech..

[34] Çağlar Y., Şener S. (2016). Size effect tests of different notch depth specimens with support rotation measurements. Eng. Fract. Mech..

[35] Grégoire D., Rojas-Solano L., Pijaudier-Cabot G. (2013). Failure and size effect for notched and unnotched concrete beams. Int. J. Numer. Anal. Methods Geomech..

[36] Hoover C.G., Bažant Z.P. (2014). Universal size-shape effect law based on comprehensive concrete fracture tests. J. Eng. Mech..

[37] Hu X., Guan J., Wang Y., Keating A., Yang S. (2017). Comparison of boundary and size effect models based on new developments. Eng. Fract. Mech..

[38] Duan K., Hu X., Wittmann F.H. (2003). Boundary effect on concrete fracture and non-constant fracture energy distribution. Eng. Fract. Mech..

[39] Duan K., Hu X., Wittmann F.H. (2006). Scaling of quasi-brittle fracture boundary and size effect. Mech. Mater..

[40] Bažant Z., Yu Q. (2009). Universal size effect law and effect of crack depth on quasi-brittle structure strength. J. Eng. Mech..

[41] Hoover C.G., Bažant Z.P. (2014). Cohesive crack, size effect, crack band and work-of-fracture models compared to comprehensive concrete fracture tests. Int. J. Fract..

[42] Mazars J. (1986). A description of micro- and macroscale damage of concrete structures. Eng. Fract. Mech..

[43] Lorentz E. (2017). A nonlocal damage model for plain concrete consistent with cohesive fracture. Int. J. Fract..

[44] Feng D.-C., Wu J.-Y. (2018). Phase-field regularized cohesive zone model (CZM) and size effect of concrete. Eng. Fract. Mech..

[45] Barbat G.B., Cervera M., Chiumenti M., Espinoza E. (2020). Structural size effect: Experimental, theoretical and accurate computational assessment. Eng. Struct..

[46] Parrilla Gómez A., Stolz C., Moës N., Grégoire D., Pijaudier-Cabot G. (2017). On the capability of the Thick Level Set (TLS) damage model to fit experimental data of size and shape effects. Eng. Fract. Mech..

[47] Zhang Y., Shedbale A.S., Gan Y., Moon J., Poh L.H. (2021). Size effect analysis of quasi-brittle fracture with localizing gradient damage model. Int. J. Damage Mech..

[48] Wosatko A., Pamin J., Winnicki A. (2018). Numerical prediction of deterministic size effect in concrete bars and beams. Computational Modelling of Concrete Structures: Proceedings of the Conference on Computational Modelling of Concrete and Concrete Structures (EURO-C 2018), Bad Hofgastein, Austria, 26 February–1 March 2018.

[49] Marzec I., Bobiński J. (2019). On some problems in determining tensile parameters of concrete model from size effect tests. Pol. Marit. Res..

[50] Havlásek P., Grassl P., Jirásek M. (2016). Analysis of size effect on strength of quasi-brittle materials using integral-type nonlocal models. Eng. Fract. Mech..

[51] Bažant Z.P., Le J.-L., Hoover C.G. Nonlocal boundary layer (NBL) model: Overcoming boundary condition problems in strength statistics and fracture analysis of quasibrittle materials. Proceedings of the 7th International Conference on Fracture Mechanics of Concrete and Concrete Structures.

[52] Vořechovský M. (2007). Interplay of size effects in concrete specimens under tension studied via computational stochastic fracture mechanics. Int. J. Solids Struct..

[53] Van Vliet M., Van Mier J. (2000). Experimental investigation of size effect in concrete and sandstone under uniaxial tension. Eng. Fract. Mech..

[54] Van Vliet M., Van Mier J. (2000). Size effect of concrete and sandstone. Heron.

[55] Melenk J.M., Babuška I. (1996). The partition of unity finite element method: Basic theory and applications. Comput. Methods Appl. Mech. Eng..

[56] Zi G., Belytschko T. (2003). New crack-tip elements for XFEM and applications to cohesive cracks. Int. J. Numer. Methods Eng..

[57] Tejchman J., Bobiński J. (2013). Continuous and Discontinuous Modelling of Fracture in Concrete Using FEM.

[58] Asferg J.L., Poulsen P.N., Nielsen L.O. (2007). A consistent partly cracked XFEM element for cohesive crack growth. Int. J. Numer. Methods Eng..

[59] Cox J.V. (2009). An extended finite element method with analytical enrichment for cohesive crack modelling. Int. J. Numer. Methods Eng..

[60] (2016). Abaqus Documentation.

[61] De Borst R. (1987). Computation of post-bifurcation and post-failure behaviour of strain-softening solids. Comput. Struct..

[62] Planas J., Sanz B., Sancho J.M. (2016). Transition from smeared to localized cracking in macro-defect-free quasibrittle structures. Procedia Struct. Integr..

[63] Syroka-Korol E., Tejchman J., Mróz Z. (2013). FE calculations of a deterministic and statistical size effect in concrete under bending within stochastic elasto-plasticity and non-local softening. Eng. Struct..

